# Biomarkers of Colorectal Cancer Risk Decrease 6 months After Roux-en-Y Gastric Bypass Surgery

**DOI:** 10.1007/s11695-017-2953-6

**Published:** 2017-10-08

**Authors:** Sorena Afshar, Fiona Malcomson, Seamus B. Kelly, Keith Seymour, Sean Woodcock, John C. Mathers

**Affiliations:** 1 Human Nutrition Research Centre, Institute of Cellular Medicine, Newcastle University, Campus for Ageing and Vitality, Newcastle on Tyne, NE4 5PL UK; 2 Northumbria Healthcare NHS Foundation Trust, North Tyneside General Hospital, Rake Lane, North Shields, NE29 8NH UK

**Keywords:** Bariatric surgery, Gastric bypass, Obesity, Colorectal cancer, Proliferation

## Abstract

**Purpose:**

The impact of weight loss on obesity-related colorectal cancer (CRC) risk is not well defined. Previous studies have suggested that Roux-en-Y gastric bypass (RYGB) surgery may have an unexpected adverse impact on CRC risk. This study aimed to investigate the impact of RYGB on biomarkers of CRC risk.

**Materials and methods:**

Rectal mucosal biopsies and blood were obtained from patients undergoing RYGB (*n* = 22) and non-obese control participants (*n* = 20) at baseline and at a median of 6.5 months after surgery. Markers of systemic inflammation and glucose homeostasis were measured. Expression of pro-inflammatory genes and proto-oncogenes in the rectal mucosa was quantified using qPCR. Crypt cell proliferation state of the rectal mucosa was assessed by counting mitotic figures in whole micro-dissected crypts.

**Results:**

At 6.5 months post-surgery, participants had lost 29 kg body mass and showed improvements in markers of glucose homeostasis and in systemic inflammation. Expression of pro-inflammatory genes in the rectal mucosa did not increase and *COX-1* expression fell significantly (*P* = 0.019). The mean number of mitoses per crypt decreased from 6.5 to 4.3 (*P* = 0.028) after RYGB.

**Conclusion:**

RYGB in obese adults led to lower rectal crypt cell proliferation, reduced systemic and mucosal markers of inflammation and improvements in glucose regulation. These consistent findings of reduced markers of tumourigenic potential suggest that surgically induced weight loss may lower CRC risk.

**Electronic supplementary material:**

The online version of this article (10.1007/s11695-017-2953-6) contains supplementary material, which is available to authorized users.

## Introduction

Obesity is a well-established risk factor for colorectal cancer (CRC) [1]. Obese individuals are estimated to have a 33% higher risk of CRC compared with those with a normal body mass index (BMI) [2]. Excess adiposity is also a significant risk factor for colorectal adenoma (CRA) [3], suggesting that it plays a role in the early stages of CRC development.

There are several plausible mechanisms through which body fatness could increase CRC risk, which have been reviewed in detail elsewhere [4]. Obese individuals exhibit a state of chronic low-grade systemic inflammation [5]. Chronic inflammation of the colorectal mucosa in individuals with inflammatory bowel disease increases CRC risk [6]. Inflammation may contribute to CRC development through increased genomic damage [7]. Furthermore, obesity leads to insulin resistance and the resultant hyperinsulinaemia is associated with increased CRC risk [8].

Bariatric surgery can result in dramatic weight loss, especially in the short to medium term [9]. In addition, obesity-related systemic inflammation improves after bariatric surgery [10, 11] and most patients achieve a reversal of insulin resistance [12]. However, the impact of intentional weight loss on subsequent CRC risk is poorly understood. To date, there have been only four observational studies that have reported the effects of bariatric surgery on subsequent CRC incidence [13–16]. Pooled analysis of data from these studies shows that bariatric surgery is associated with a significantly (*P* = 0.004) lower CRC incidence (RR 0.73, 95% CI: 0.58–0.90) [17].

However, some investigations of surrogate biomarkers of CRC risk following bariatric surgery suggest that the opposite may be true, at least for some types of bariatric surgery. Sainsbury et al. studied obese patients who underwent Roux-en-Y gastric bypass (RYGB) surgery and normal BMI controls [10]. Rectal biopsy samples were collected before and 6 months after surgery. Before surgery, the obese patients had a higher rectal epithelial cell mitosis count (increased by 73%, *P* < 0.01), higher crypt area (increased by 36%, *P* < 0.01) and crypt branching was more than twice as common when compared with controls. These changes are associated with higher CRC risk and are some the earliest changes seen in humans predisposed to gastrointestinal cancer [18]. However, unexpectedly, after RYGB with the resultant significant weight loss, there was a further increase in mitosis (75% higher than pre-surgery, *P* = 0.001) and a decrease in apoptosis (*P* = 0.033). This was accompanied by a greater expression of pro-inflammatory genes (*COX-1*, *COX-2* and *IL-6*) at the mRNA level within the rectal mucosa. The authors concluded that the hyper-proliferative state after RYGB may be associated with an increased long-term risk of CRC [10]. Importantly, follow-up of the same RYGB patients at 3 years showed sustained elevation of rectal epithelial cell proliferation and crypt size and raised expression of the pro-tumorigenic cytokine macrophage migration inhibitory factor (*MIF*) in the mucosa [19]. A plausible mechanistic case was made that ‘malabsorptive’ bariatric surgery such as RYGB could have adverse effects on the colorectal epithelium and, therefore, on CRC risk because of the diversion into the colon of damaging luminal content [10]. Such an effect would not be anticipated with ‘restrictive’ bariatric surgery, where the normal processes of small bowel digestion and absorption are largely unaffected. This hypothesis is supported by the finding that the sleeve gastrectomy (SG) does not lead to increased mucosal biomarkers of CRC risk [20].

The overall aim of this project was to assess the impact of RYGB on biomarkers of CRC risk. Specifically, we tested the hypothesis that surgically induced weight loss results in lower rectal mucosal crypt cell proliferation, reflecting an overall reduction in CRC risk.

## Materials and Methods

### Recruitment

#### Bariatric Surgery Patients

**Fig. 1:**
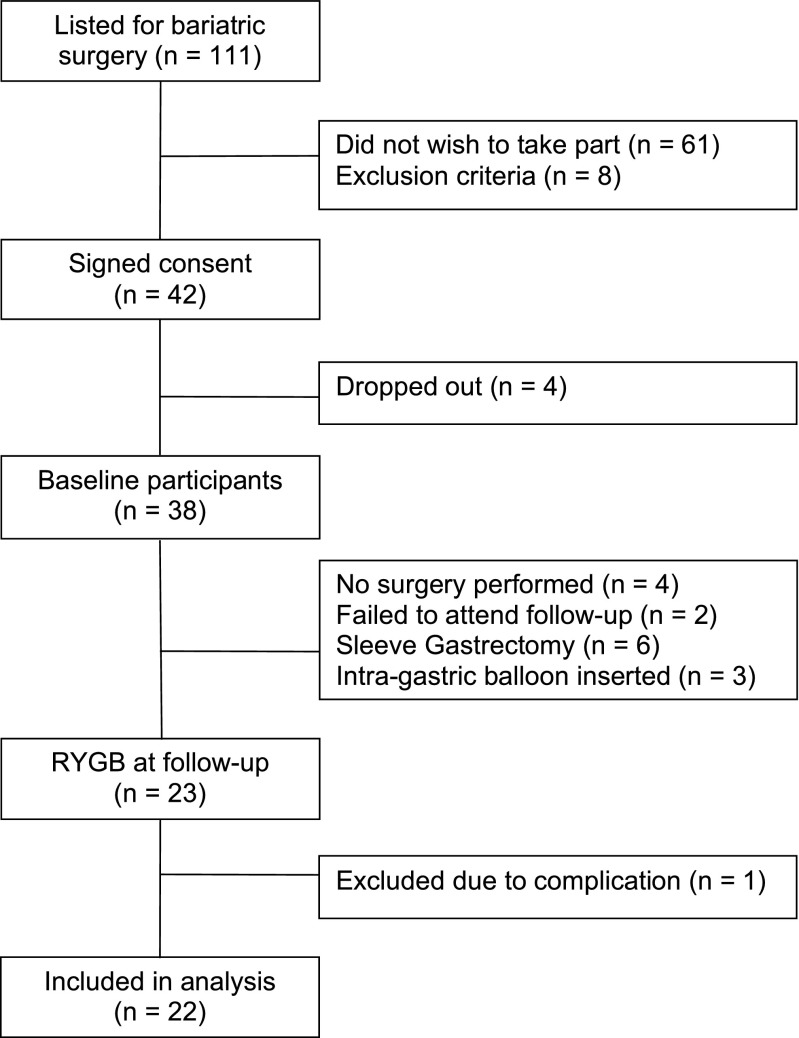
Flow of participants through the study

We recruited adults (18–65 years old) listed for bariatric surgery at a single centre (North Tyneside General Hospital, UK) from November 2013 to November 2014. All patients listed for a bariatric surgery during this period were approached by the research team after their pre-operative clinic visit (Fig. 1). All bariatric surgery candidates had to complete a 12-week multi-disciplinary weight management programme and to achieve at least 5% body weight reduction. Exclusion criteria included: previous bariatric surgery (*n* = 5), oral anticoagulation (*n* = 2) and use of immunosuppressive therapy (*n* = 1). Patients who had a SG (*n* = 6) or an intra-gastric balloon inserted (*n* = 3) were excluded from this analysis. RYGB involved laparoscopic formation of a 50-ml gastric pouch with a 100–150 cm alimentary limb and 60–75 cm biliopancreatic limb. None of the participants had a concurrent cholecystectomy.

#### Non-obese Control Participants

Non-obese (BMI < 30 kg/m^2^) patients who had either a flexible sigmoidoscopy or colonoscopy within the past year which did not show any significant colorectal pathology (CRC, CRA or active inflammation) were invited to take part at least 1 month after their endoscopic examination to allow washout of the effects of bowel preparation. We also used rectal biopsies from eight healthy non-obese participants at baseline from a previous dietary intervention study (DISC study www.clinicaltrials.gov/NCT01214681) who were recruited from the same gastroenterology clinics and using identical biopsy protocols [21].

### Study Design

The participants’ journey and study visits are detailed in (Supplementary Fig. 1). Bariatric surgery and control participants had assessment and sample collection at baseline. The bariatric surgery patients were invited for follow-up at around 6 months post-surgery.

### Anthropometry

Demographic data, as well as medical history and current medications, were recorded during a face-to-face interview with a researcher (SA) in a hospital setting and supplemented by a review of medical records. Anthropometric measurements, including height, weight, waist and hip circumference, were made using a standardised protocol by a single observer (SA) at the time patients were listed for surgery. Percentage body fat was estimated using bioimpedance scales (Tanita TBF-300MA body composition analyser).

### Systemic Markers

Venepuncture was performed after a standardised 6 hour fast. Blood was collected in BD Vacutainer Plus plastic serum tubes and centrifuged at 3100 g for 5 minutes within 20 minutes of collection. Serum was aspirated, frozen immediately and stored at –80 °C for subsequent batch analysis. Plasma from a potassium oxalate/sodium fluoride-containing tube was collected for fasting glucose measurement using the oxidase method. High sensitivity C-reactive protein (hsCRP) was quantified on an autoanalyser (Roche-Hitachi Modular P, Roche Diagnostics, Germany). Serum insulin and leptin were measured using multiplex assay kit (K15164C-1, MSD, USA). High-performance liquid chromatography (Tosoh HLC-723G8 glycohaemoglobin analyser, Tosoh Bioscience) was used for quantification of Haemoglobin A1c (HbA1c). Homeostasis model assessment-insulin resistance (HOMA-IR) was calculated using the HOMA2 calculator v2.2.3 [22].

### Rectal Mucosal Biopsies

No bowel preparation was used prior to taking rectal biopsies, because both oral and enema bowel preparation has been shown to have a significant impact on crypt cell proliferation in the rectal mucosa [23, 24]. Macroscopically normal rectal mucosa at a distance of 10 cm from the anal verge was biopsied. One biopsy was fixed in 10% formalin and examined by a histopathologist; one in Carnoy’s solution (70% ethanol, 30% acetic acid) for a minimum of 2 h, then transferred to 70% ethanol and stored at 4 °C for subsequent crypt cell proliferation state (CCPS) analysis; one in RNAlater solution (Ambion, Texas, USA) and stored at −80 °C for gene expression analysis.

### Crypt Cell Proliferation State

CCPS was assessed by counting the number of mitoses per crypt after whole crypt microdissection (WCMD) [25]. Increased proliferation in the basal part of the crypt and expansion of this proliferative compartment to the upper parts of the crypt are some of the earliest changes seen in humans predisposed to gastrointestinal cancer [18]. All samples used for this analysis were re-labelled and the assessor was blinded to the identity of each sample. Carnoy’s fixed rectal biopsies were rehydrated and stained using Schiff’s reagent before WCMD was performed as described [18], using a microscope (Olympus SZ40) at ×25 magnification.

Ten randomly selected non-branching dissected crypts were analysed for each biopsy. A compound microscope (Olympus BX51) was used to examine the slides at ×40 magnification with a calibrated eyepiece graticule to make measurements. Crypt area was estimated, based on the assumption of cylindrical shape. Mitotic figures were identified by focusing through all the layers of the crypt. Cells in late prophase, metaphase, anaphase and early telophase were recorded as mitotic. Crypts were divided into ten equal length compartments and the number of mitotic figures in each compartment was recorded for each of the ten crypts examined. The presence of at least one branching or bifid crypt was recorded.

Assessments were carried out by two assessors (SA and FM). Ten samples were randomly selected for the reproducibility analysis with each of the ten crypts per sample analysed in tandem. Intra-class correlation coefficient (ICC) analysis showed excellent agreement between the two raters (mitoses per crypt count ICC 0.99, 95% CI 0.95–0.99, *P* < 0.001; crypt length ICC 0.97, 95% CI 0.87–0.99, *P* < 0.001; crypt width ICC 0.98 0.95 to 0.99, *P* < 0.001). In addition, assessors quantified CCPS and crypt dimensions for both pre- and post-surgery for any individual study participant.

### Expression of Pro-inflammatory Genes and Proto-oncogenes

RNA was extracted from rectal biopsies using the Qiagen miRNeasy mini kit (Qiagen, UK). Concentration and purity of RNA were checked using the NanoDrop 1000 spectrophotometer (Thermo Scientific) and integrity using agarose gel electrophoresis. cDNA was synthesised using the RT^2^ First Strand kit (Qiagen, UK). RT^2^ primer assays and SYBR Green ROX qPCR Mastermix (Qiagen, UK) were used to quantify expression of the pro-inflammatory genes *IL-6*, *MIF*, *COX-1* and *-2* and the proto-oncogenes *c-FOS* and *c-JUN* at the mRNA level using the Applied Biosystems® StepOnePlus™ real-time PCR machine. Expression was quantified using the delta Ct approach with *GAPDH* and *18S* rRNA used as reference genes [26].

### Statistical Analysis

Statistical analysis was carried out using SPSS software (Version 22.0 for Windows, SPSS, Chicago, USA). Data are reported as mean ± standard error or median and interquartile range (IQR) for normally and non-normally distributed data, respectively. Paired sample or independent sample *t* tests were used to analyse normally distributed data, as appropriate. For non-normally distributed data, Wilcoxon signed-rank and Mann-Whitney tests were used, as appropriate. Cross-tabulation was carried out using Fisher’s exact test or McNemar’s test for dichotomous variables. Statistical significance was set at *P* < 0.05.

## Results

Twenty-three RYGB patients eligible for follow-up attended a study visit at a median of 6.5 months (range 5.8 to 7.8) after surgery. One of these participants had significant post-operative complications after a RYGB with an anastomotic leak requiring total parenteral nutrition and was therefore excluded from this analysis. Participant characteristics, anthropometry and clinical outcomes are shown in Table 1.

**Table 1:** Participant characteristics at baseline and post-RYGB

	Non-obese control (*N* = 20)	Obese pre-surgery (*N* = 22)	Obese post-surgery (*N* = 22)	*P* value control vs. pre-surgery	*P* value pre- vs. post-surgery
Age (years)*	46.0 (2.6)	47.0 (1.2)	–	0.720	–
Sex—*N* (%) female	12 (60)	4 (18)	–	0.175†	–
Smoking—*N* (%)					
Daily	5 (25)	0	1 (5)	0.002†	< 0.001‡
Occasional	1 (5)	0	11 (52)		
Ex-smoker	2 (10)	11 (50)	9 (43)		
Never smoked	12 (60)	10 (45)	0		
Missing data	0	1 (4)	0		
NSAID use—*N* (%)	5 (25)	10 (45)	1 (5)	0.209†	0.004^§^
Previous cholecystectomy—*N* (%)	2 (10)	4 (18)	–	0.665†	–
Weight (kg)*	71.8 (2.8)	114.8 (3.7)	86.3 (3.5)	< 0.001	< 0.001
BMI (kg/m^2^)*	25.4 (0.5)	42.4 (1.4)	31.3 (1.2)	< 0.001	< 0.001
Body fat (%)*	30.3 (1.3)	47.6 (1.0)	36.1 (1.5)	< 0.001	< 0.001
Waist (cm)*					
Men	95.9 (2.9)	137.3 (2.0)	112.5 (4.5)	< 0.001	0.007
Women	83.4 (2.2)	117.5 (2.2)	91.9 (3.5)	< 0.001	< 0.001
Waist to hip ratio*					
Men	0.93 (0.01)	1.07 (0.03)	0.99 (0.03)	0.001	0.067
Women	0.82 (0.02)	0.89 (0.01)	0.84 (0.02)	0.010	0.007

Independent sample *t* test used to compare non-obese control and obese pre-surgery participants, unless otherwise indicated. Paired sample *t* test used to compare participants pre- and post-surgery, unless otherwise indicated

*NSAID* non-steroidal anti-inflammatory agent

*Values indicate mean (SEM)

†Fisher’s exact test

‡Wilcoxon sign test

§Related sample McNemar test

At baseline, the obese and non-obese groups were well matched with no differences in age, sex, NSAID use and previous cholecystectomy rates. However, none of the obese pre-surgery participants reported being current smokers whilst 30% of the non-obese control participants reported being current smokers (daily/occasional). Non-smoking was a strict selection criterion by the clinical team for bariatric surgery candidates. As anticipated, all measures of body fatness were significantly higher for obese compared with non-obese participants. At 6.5 months post-surgery, participants had lost 29 kg body mass, the majority of which (mean 23 kg) was body fat.

### Systemic Markers Indicate Improvements in Inflammation and Insulin Resistance After Bariatric Surgery

Pre-surgery, mean serum hsCRP concentration was higher, but not significantly so, in the obese group compared with the non-obese controls (Table 2). Following surgically induced weight loss, hsCRP concentration fell by 71% (*P* < 0.001). As expected [27], fasting glucose and serum insulin, leptin and HOMA-IR were all significantly elevated in the obese group pre-surgery compared with non-obese controls, although the difference in HbA1c and did not reach statistical significance (Table 2). At baseline, serum leptin concentration was nearly six times higher in obese patients compared with non-obese controls and decreased significantly after bariatric surgery (Table 2). Moreover, surgically induced weight loss resulted in significant improvements in all measured markers of glucose homeostasis (Table 2).

**Table 2:** Systemic markers of inflammation, glucose homeostasis and adiposity

Marker	Non-obese control (*N* = 12)	Obese pre-surgery (*N* = 22)	Obese post-surgery (*N* = 22)	P value control vs. pre-surgery	P value pre- vs. post-surgery
hsCRP (mg/L)	3.6 (1.2)	5.5 (0.9)	1.6 (0.4)	0.190	< 0.001
Fasting glucose (mmol/L)	4.5 (0.1)	5.8 (0.4)	4.9 (0.4)	0.003	0.001
HbA1c (mmol/mol)	36.1 (0.9)	42.2 (2.9)	38.5 (2.4)	0.194	0.001
Insulin (pmol/L)	64.6 (10.6)	117.1 (19.6)	54.3 (7.8)	0.025	0.001
HOMA-IR	1.2 (0.2)	2.2 (0.3)	1.0 (0.1)	0.014	< 0.001
Leptin (ng/mL)	11.2 (3.2)	62.5 (14.3)	15.6 (3.9)	0.002	0.003

Data are presented as mean (SEM). Independent sample *t* test used to compare non-obese control and obese pre-surgery participants. Paired sample *t* test used to compare participants pre- and post-surgery

*hsCRP* highly sensitive C-reactive protein, *HbA1c* haemoglobin A1c, *HOMA-IR* homeostasis model assessment-insulin resistance

### Expression of Pro-inflammatory Genes and Proto-oncogenes in the Rectal Mucosa After Bariatric Surgery

There was no evidence of macroscopic inflammation of the rectal mucosa in any of the participants and none of the rectal biopsies showed evidence of microscopic colitis when examined by a consultant histopathologist. At baseline, rectal mucosal expression of the pro-inflammatory genes, *COX-1*, *COX-2*, *IL-6* and *MIF*, was similar in both obese and non-obese groups (Table 3). After bariatric surgery, *COX-1* expression in rectal biopsies fell significantly (*P* = 0.019) but there was no change in expression of *COX-2*, *IL-6* and *MIF* (Table 3). There were no differences in expression of the proto-oncogenes *c-FOS* and *c-JUN* between non-obese and obese participants and no changes following bariatric surgery in the obese (Table 3).

**Table 3:** Expression of pro-inflammatory genes in rectal biopsies from non-obese control participants and obese participants pre- and post-RYGB

Gene	Non-obese control (*N* = 20)	Obese pre-surgery (*N* = 22)	Obese Post-surgery (*N* = 22)	*P* value control vs. pre-surgery	*P* value pre- vs. post-surgery
*MIF*	2.162 (2.375)	1.796 (1.085)	2.239 (1.269)	0.497	0.322
*COX-1*	0.038 (0.021)	0.042 (0.029)	0.031 (0.015)	0.420	0.019
*COX-2*	0.012 (0.089)	0.009 (0.004)	0.010 (0.008)	0.676	0.931
*IL-6*	0.007 (0.037)	0.003 (0.005)	0.006 (0.010)	0.068	0.322
*c-FOS*	0.187 (0.230)	0.128 (0.321)	0.169 (0.665)	0.548	0.306
*c-JUN*	0.097 (0.109)	0.105 (0.057)	0.101 (0.096)	0.450	0.638

Data are expressed as median (IQR) 2^-ΔCT^ × 1000 relative to geometric mean of reference genes *GAPDH* and *18S* rRNA. Mann-Whitney U test used to compare non-obese control and obese pre-surgery participants. Wilcoxon signed-rank test used to compare participants pre- and post-surgery

### Rectal Crypt Cell Proliferation Decreases After Bariatric Surgery with Concomitant Changes in Distribution of Mitotic Figures

**Fig. 2:**
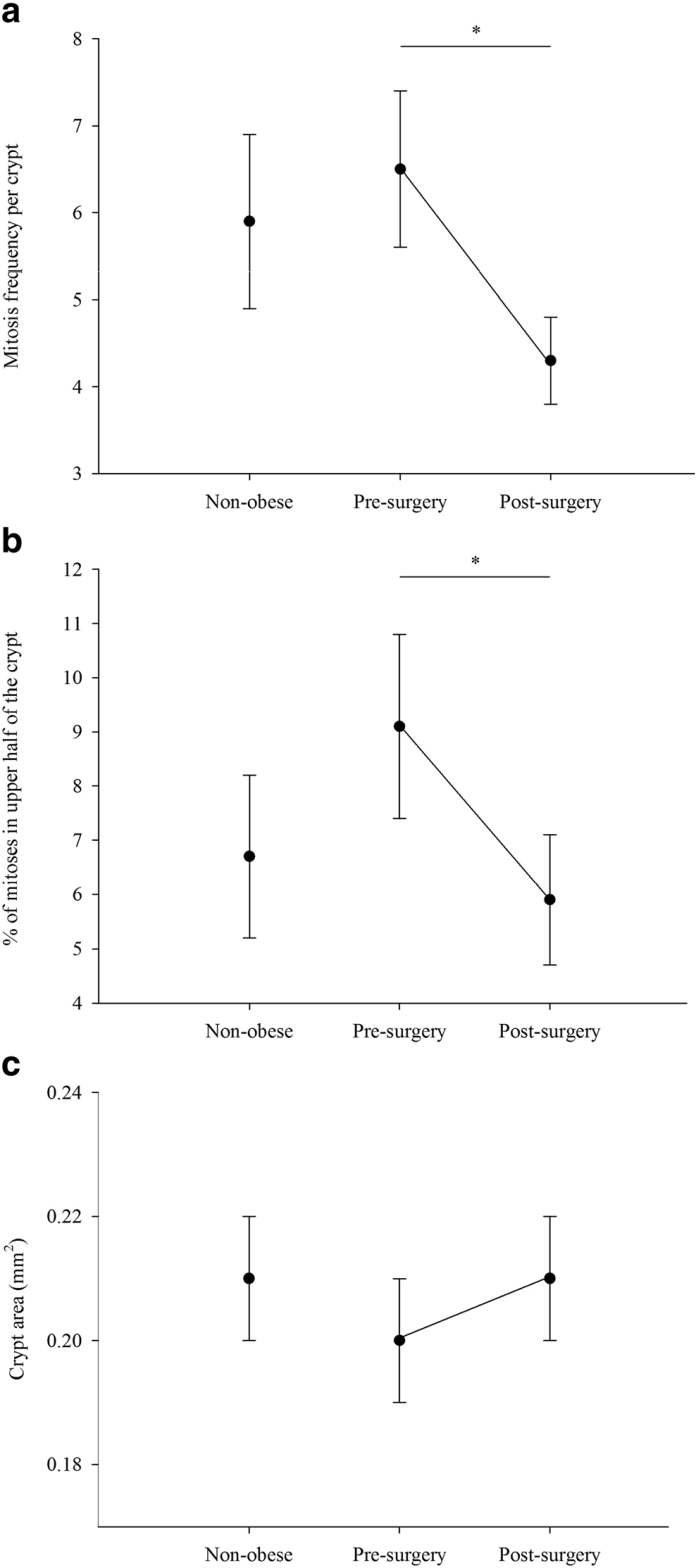
Rectal mucosal crypt cell proliferation status in non-obese control participants and in obese participants pre- and post-RYGB surgery. **a** Mean total number of mitoses per crypt. **b** Percentage of mitoses in the upper half of the crypt. **c** Mean crypt area. Dark circles represent mean for the group and error bars represent standard error. Independent sample *t* test used to compare non-obese control and obese pre-surgery participants. Paired sample *t* test used to compare obese participants pre- and post-surgery (*p < 0.05)

Crypt cell kinetics and crypt area were similar in obese patients at baseline and in non-obese participants (Table 4 and Fig. 2a–c). However, after bariatric surgery, the mean number of mitoses per crypt decreased significantly (34% decrease) and the proportion of mitoses in top half of the crypt fell by 35% (Fig. 2a, b). Despite these substantial changes in measures of cell proliferation, there was no significant change in crypt area after surgery (Fig. 2c). There was also no significant difference in the number of participants in whom branching crypts were detected; two patients had branching crypts pre-surgery and five post-surgery (*P* = 0.375).

**Table 4:** Rectal mucosal crypt cell proliferation state and crypt dimensions

	Non-obese control (*N* = 20)	Obese pre-surgery (*N* = 22)	Obese post-surgery (*N* = 22)	*P* value control vs. pre-surgery	*P* value pre- vs. post-surgery
Total mitoses per crypt	5.9 (1.0)	6.5 (0.9)	4.3 (0.5)	0.640	0.028
Mitoses in top half of the crypt (%)	6.7 (1.5)	9.1 (1.7)	5.9 (1.2)	0.199	0.047
Crypt length (mm)	0.53 (0.01)	0.53 (0.02)	0.55 (0.01)	0.591	0.448
Crypt width (mm)	0.12 (0.003)	0.11 (0.004)	0.12 (0.002)	0.360	0.212
Crypt area (mm^2^)	0.21 (0.01)	0.20 (0.01)	0.21 (0.01)	0.786	0.282

Data are presented as mean (SEM). Independent sample *t* test used to compare non-obese control and obese pre-surgery participants. Paired sample *t* test used to compare participants pre- and post-surgery

## Discussion

Our main findings were a 34% decrease in the mean number of mitoses per crypt and a contraction of the crypt proliferative compartment at 6.5 months after RYGB. These changes were associated with dramatic improvements in systemic markers of glucose homeostasis and inflammation. There was also a decrease in expression of the pro-inflammatory gene *COX-1* in the rectal mucosa. Furthermore, there were no significant changes in the expression of pro-tumourogenic (*MIF*, *c-FOS* and *c-JUN*) or pro-inflammatory genes (*COX-2* and *IL-6*) in the rectal mucosa. Together, these observations suggest that surgically induced weight loss may reduce obesity-related risk of CRC.

### Findings in the Context of Other Studies

#### Does Obesity Alter Crypt Cell Proliferation in the Human Colorectum?

There is paucity of research on the impact of obesity on CCPS in humans but the limited evidence available suggests a higher proliferation state in the rectal mucosa of obese compared with normal BMI individuals [10, 28]. On this basis, the lack of a difference in CCPS measures between obese and non-obese individuals in our study is unexpected but may be due to the fact that the BMI of our obese patients was considerably lower than that reported by previous studies in this field [10, 28]. The inclusion of smokers in the non-obese control group may also have been contributing factor. However, a sensitivity analysis excluding smokers from the non-obese control group did not result in any significant difference in CCPS measures between obese and non-obese groups. Also, smoking status was not a significant factor in the expression of any of the genes of interest (Kruskal-Wallis test).

#### Effects of Bariatric Surgery on Rectal Crypt Cell Proliferation

Sainsbury et al. found a significantly higher number of mitoses per crypt in the rectum of patients 6 months after RYGB compared with baseline [10]. In contrast, a similar study by Kant et al., from the same laboratory, found no significant change in rectal mucosal CCPS 6 months after a SG compared with baseline [28]. Key characteristics of the participants in, and findings from, these two studies and from the present study are summarised in Table 5.

**Table 5:** Studies of the impact of bariatric surgery on rectal CCPS at 6 months post-surgery

	Present study (*N* = 22)	Sainsbury et al. [10] (*N* = 24)	Kant et al. [20] (*N* = 23)
Procedures	RYGB	RYGB	SG
BMI (kg/m^2^)			
Baseline	42.4	54.4	65.7
Follow-up	31.3	41.8	50.1
RYGB limb lengths			
BP limb	63	150	n/a
Roux limb	127	150	n/a
Concurrent cholecystectomy (%)	0	46	NR
CRP (mg/l)			
Baseline	5.5	8.7	17.5
Follow-up	1.6	3.8	13.5
Gene expression			
*COX-1*	↓	↑	↔
*COX-2*	↔	↑	↔
*IL-6*	↔	↑	↔
*MIF*	↔	↔	↑
Mitoses per crypt	↓	↑	↔
Mitoses in upper part of crypt	↓	↑	↔
Crypt area	↔	↔	↔
Branching crypts	↔	↔	↔

*NR* not reported

Although many aspects of the study design used by Sainsbury et al. [10] and in the present study were similar, there are several key features of the patient population and of the surgical procedures which differed between the studies and which may have contributed to contrasting outcomes. First, the pre-surgery mean BMI of the patients studied by Sainsbury et al. was 12 kg/m^2^ higher than that of our patients. The mean post-surgery BMI of Sainsbury’s patients was similar to the pre-surgery BMI of our patients. However, more pertinently, in the Sainsbury et al. study, RYGB procedures were performed with longer limb lengths (roux limb 150 cm, BP limb 150 cm) than in the present study. BP limbs of 150 cm are significantly longer than usual current surgical practice. The most recent National Bariatric Surgery Registry (NBSR) report [29] of bariatric procedures performed in the UK and Ireland shows that the vast majority of RYGB procedures were carried out with a BP limb length of ≤ 100 cm and only 3.8% had a BP limb of 150 cm. Longer roux and BP limbs have been associated with an increase in malabsorptive complications, including an increase in diarrhoea [30, 31]. In this study cohort, we have previously reported that diarrhoea, a common symptom of malabsorption, was rare post-surgery [32]. Therefore, we propose that the limb lengths used in the present study are unlikely to cause significant malabsorption [32]. We hypothesise that longer bypass limbs are more likely to cause hyper-proliferation through malabsorption and exposure of the colorectum to harmful luminal content. This hypothesis is supported by the increase in rectal mucosal expression of pro-inflammatory genes (*COX-1*, *COX-2* and *IL-6*) in the Sainsbury et al. study, which is in stark contrast with the present study, as well as the SG study by Kant et al. (Table 5).

Another notable difference between studies is the performance of concurrent cholecystectomy in almost half of the patients in the study by Sainsbury and colleagues (Table 5). Others have shown that the mitotic index of colonic mucosa (biopsies collected 20 cm from the anal orifice) increased by approximately 50% at 6 months after cholecystectomy [33], and cholecystectomy has been associated with increased CRC risk [34–36]. Faecal secondary bile acids, associated with both increased cell proliferation and increased CRC risk, are also raised after cholecystectomy [37, 38].

#### The Effects of Weight Loss Through Lifestyle Intervention on Rectal Crypt Cell Proliferation

Using autoradiography after incubation of tissue with ^3^H-thymidine, Steinbach et al. measured rectal mucosal cell proliferation before and after a 16-week weight loss intervention with caloric restriction in a group of adults with initial mean BMI 38 kg/m^2^ [39]. This intervention produced a fall of 8.6% in body weight, 39% lower whole crypt labelling index and 57% reduction in upper crypt labelling. These changes in rectal cell kinetics are similar to our observations, despite a more modest degree of weight loss in the lifestyle intervention study.

#### Potential Mechanisms for the Beneficial Effects of Weight Loss on CRC Risk

##### Improvement in Hyperinsulinaemia

Obesity is strongly associated with hyperinsulinaemia which is mechanistically linked to CRC [4]. Hyperinsulinaemia increases the bioavailability of circulating insulin-like growth factor-1 (IGF-1) by inhibiting production of IGF-binding proteins [4]. IGF-1 binds to the IGF-1 receptor, which is expressed in normal colonic tissue [40], and induces a signalling cascade which leads to cell growth, proliferation and inhibition of apoptosis [41]. We propose that the improvements in hyperinsulinaemia after surgically induced weight loss (Table 2) may contribute to the changes in CCPS we observed after bariatric surgery.

##### Reduced Systemic and Gut Inflammation

We found significant improvement in the obesity-related low-grade systemic inflammatory state post-surgery (Table 3). Furthermore, *COX-1* expression in the rectal mucosa was reduced significantly with no significant change in the expression of other pro-inflammatory genes (*MIF*, *COX-2* and *IL-6*). Importantly, the use of NSAID medication was less frequent post-surgery (Table 1), which makes it unlikely that NSAID use was a confounder.

Others have shown that 10% weight loss induced by a very-low-calorie diet reduced circulating concentrations of inflammatory cytokines (TNF-α, IL-1β, IL-8 and MCP-1) by 25–57% in rectosigmoid biopsies and reduced T cell and macrophage counts [42]. Faecal calprotectin (FCP), an established marker of whole gut inflammation, is positively correlated with obesity, as well as with other lifestyle factors associated with CRC risk [43]. One fifth of overweight/obese individuals enrolled in a weight loss programme (Slimming World) had a high FCP (> 50 μg/g) at baseline and, in these individuals, FCP reduced during the study period [44]. These studies are in keeping with the hypothesis that weight loss in overweight/obese individuals reduces both systemic and gut inflammation. Given that inflammation is an enabling characteristic in tumorigenesis [45], it is plausible that reducing inflammation through weight loss may reduce CRC risk.

##### Exposure to Bile Acids

Bariatric surgery, especially the RYGB, disrupts the enterohepatic bile circulation. A systematic review found seven studies reporting on the effects of RYGB on fasting systemic bile acids (BAs), six of which showed higher systemic BAs after RYGB [46]. This is important as serum deoxycholic acid concentrations are positively correlated with colonic mucosal proliferation [47]. However, little is known about the impact of modern bariatric procedures on faecal BAs. Since shortening of effective small bowel and the resultant intractable diarrhoea are associated with increased concentrations of BAs in the colon [48], it is likely that bariatric procedures with significant shortening of small bowel length will have a similar outcome.

Historical context comes from studies of the jejunoileal bypass (JIB), a procedure popular in 1960/1970s, which induced malabsorption by bypassing more than 90% of the small bowel and creating a short bowel syndrome. This procedure led to severe metabolic complications, including diarrhoea and life-threatening malnutrition so that up to 33% of patients who had the procedure had to have a reversal [49]. Several rat studies showed increased CRC in JIB compared with sham operated controls [50–52]. Importantly, JIB induced hyper-proliferation of the rectal mucosa in humans [53, 54] and expansion of the proliferative compartment [54] which were associated with higher concentrations of faecal BAs and lipids [54, 55].

### Strengths and Limitations

The main strength of this study is the use of paired rectal mucosal tissue in unprepared bowel from the same individuals before and after RYGB surgery, as well as in non-obese control participants. The main limitation of this analysis is the relatively small number of participants, although this is comparable to other similar studies in the field (Table 5). Whilst we could detect differences in CCPS measures from before to after RYGB surgery, we lacked statistical power to perform potentially interesting sub-group analysis.

In summary, RYGB in obese adults led to a decrease in rectal crypt cell proliferation, reduced systemic and mucosal markers of inflammation, as well as improvements in glucose regulation. These findings suggest that such surgically induced weight loss may lower CRC risk, in keeping with the limited evidence of protection against CRC which has been reported in observational studies [17]. We hypothesise that RYGB involving longer bypass limbs may cause hyper-proliferation through malabsorption and exposure of the colorectum to BAs and, possibly, other luminal agents. In the absence of significant specific benefits of ‘long limb’ RYGB and the potential increase in biomarkers of future CRC risk associated with this procedure, ‘long limb’ RYGB surgery should be approached with caution. Preference should be given to RYGB procedures with shorter bypass limbs which, in the present study, were associated with reduced biomarkers of CRC risk.

## Electronic supplementary material

Supplementary Figure 1 (DOCX 112 kb)
