# Supplementary figures and images for: Biomarkers of Colorectal Cancer Risk Decrease 6 months After Roux-en-Y Gastric Bypass Surgery
Source: Obes Surg. 2017 Oct 8;28(4):945–54. doi: 10.1007/s11695-017-2953-6 (PMC5880852; doi:10.1007/s11695-017-2953-6)

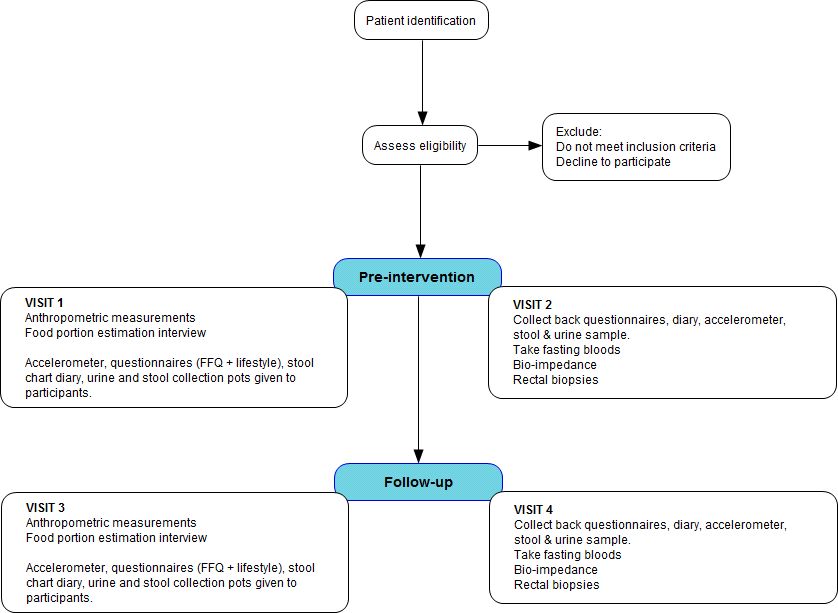


Supplementary figure 1. Study participants’ journey.

Supplement: Supplementary file 1 — (DOCX 112 kb) [file 11695_2017_2953_MOESM1_ESM.docx]
